# Outcomes of percutaneous coronary intervention in COVID-19-positive acute coronary syndrome patients: a retrospective study in Vietnam

**DOI:** 10.3389/fcvm.2025.1563415

**Published:** 2025-07-10

**Authors:** Duy Cao Phuong Le, Nguyet Thi Minh Nguyen, Quan Duy Vo

**Affiliations:** ^1^ Department of Cardiovascular Intervention, Nguyen Tri Phuong Hospital, Ho Chi Minh City, Vietnam; ^2^ Faculty of Medicine, Nguyen Tat Thanh University, Ho Chi Minh City, Vietnam

**Keywords:** COVID-19, STEMI, percutaneous coronary intervention, acute coronary syndrome, outcomes

## Abstract

**Background:**

Coronavirus disease 2019 (COVID-19) has disrupted the management of acute coronary syndrome (ACS), with emerging evidence suggesting increased complications and mortality among patients undergoing percutaneous coronary intervention (PCI). However, data from low- and middle-income settings such as Vietnam remain limited. This study aimed to evaluate the clinical characteristics and outcomes of ACS patients with COVID-19 undergoing PCI at a tertiary hospital in Vietnam.

**Methods:**

This retrospective cohort study was conducted at a tertiary hospital in Ho Chi Minh City from 2019 to 2022. Adult patients diagnosed with ACS who underwent PCI were included and stratified by COVID-19 status confirmed via RT-PCR. All patients received standard guideline-directed therapy, including dual antiplatelet and anticoagulant regimens, and were followed for 1 year to assess clinical outcomes.

**Result:**

A total of 118 patients were included, comprising 26 COVID-19-positive and 92 COVID-19-negative individuals. Baseline characteristics and cardiovascular risk factors were generally comparable between the two groups. While procedural success rates were similar, COVID-19-positive patients demonstrated higher thrombus burden and significantly increased rates of ICU admission, prolonged hospitalization, and MACCE at all timepoints. COVID-19 severity, cardiogenic shock, and multivessel disease emerged as independent predictors of adverse outcomes.

**Conclusion:**

In this Vietnamese cohort, COVID-19 infection was associated with worse clinical outcomes following PCI for ACS. These findings highlight the need for early risk stratification and resource planning during pandemic conditions. However, the small sample size, single-center design, and observational nature of the study limit its generalizability, and causal inferences should be drawn with caution.

## 1 Introduction

Coronavirus disease 2019 (COVID-19), caused by severe acute respiratory syndrome coronavirus 2 (SARS-CoV-2), emerged as a global health crisis in late 2019 and was declared a pandemic by the World Health Organization (WHO) in March 2020 (1). Since then, it has placed unprecedented strain on healthcare systems worldwide, reshaping the management of both acute and chronic conditions. In particular, care for patients with acute coronary syndrome (ACS) has been disrupted, affecting every stage of diagnosis and treatment, compromising outcomes and straining established protocols (2).

Percutaneous coronary intervention (PCI) remains the gold standard for treating ACS, particularly in ST-segment elevation myocardial infarction (STEMI). However, emerging evidence indicates that ACS patients with concurrent COVID-19 infection experience worse in-hospital outcomes, including elevated thrombus burden, higher rates of cardiogenic shock, prolonged hospitalization and increased mortality (3, 4). These adverse outcomes have been attributed to delays in care, the diversion of critical care resources, and the systemic inflammatory and prothrombotic effects of COVID-19 (5, 6).

While studies from high-income countries have begun to characterize the interplay between COVID-19 and ACS outcomes, data from low- and middle-income countries, including Vietnam, remain limited. Vietnam's healthcare system, with its centralized tertiary hospitals and constrained critical care capacity during pandemic surges, may present unique challenges in maintaining standard ACS care (7). Moreover, the distinct characteristics of the Vietnamese population such as younger average age and differing cardiovascular risk profiles could modulate disease presentation and outcomes (8).

Understanding how COVID-19 infection and the evolving pandemic landscape influenced ACS management and outcomes is essential for improving future preparedness, particularly in resource-limited settings. Specifically, the effects of pandemic waves, COVID-19 severity, and vaccination coverage on procedural characteristics and major adverse cardiac and cerebrovascular events (MACCE) remain underexplored in Vietnam.

This study aims to evaluate the clinical features, procedural outcomes, and MACCE rates among ACS patients undergoing PCI during the COVID-19 pandemic in a Vietnamese tertiary center. By identifying predictors of poor outcomes, we aim to provide insight relevant to both local clinical decision-making and national care strategies in resource-limited settings.

## 2 Material and method

### 2.1 Design of the study

This retrospective descriptive study was carried out at a tertiary care hospital in Ho Chi Minh City, Vietnam, during the COVID-19 pandemic, spanning January 2020 to December 2022. Ethical approval for the study was obtained from the hospital's Ethical Review Board.

### 2.2 Study population

The study population consisted of adult patients diagnosed with ACS who underwent PCI, with or without a positive COVID-19 result confirmed by polymerase chain reaction (PCR). All participants underwent thorough evaluations, including medical history documentation, clinical examinations, laboratory tests, cardiac biomarker analyses, and echocardiography upon admission. The diagnosis of ACS was established according to the Fourth Universal Definition of Myocardial Infarction (9).

### 2.3 Catheterization procedure

Percutaneous coronary interventions were performed in accordance with the European Society of Cardiology (ESC) guidelines (10). Before the procedure, patients received loading doses of aspirin (342 mg) and an adenosine diphosphate (ADP) receptor inhibitor (either clopidogrel 600 mg or ticagrelor 180 mg). Intravenous unfractionated heparin (UFH) was administered during the procedure. Coronary angiography was conducted through the radial or femoral artery to identify the culprit lesion, which was subsequently crossed with an angioplasty guidewire. Once the lesion was successfully crossed, predilation was performed at the operator's discretion using a semi-compliant balloon. Drug-eluting stents (DES) were deployed, with postdilation carried out as needed to ensure optimal stent apposition. Stent size and length were selected based on angiographic assessment. Following PCI, patients were monitored in the Cardiovascular Intervention Department and discharged upon maintaining hemodynamic stability for at least 24 h. Postprocedure anticoagulation therapy, including UFH or lowmolecular-weight heparin (LMWH), was provided at the operator's discretion.

### 2.4 Follow-up

After discharge, patients were prescribed dual antiplatelet therapy, consisting of daily aspirin (81 mg) combined with clopidogrel (75 mg/day) or ticagrelor (90 mg twice daily), for a minimum of 12 months, provided no contraindications were present. Additional medications, including statins, beta-blockers, angiotensin-converting enzyme inhibitors (ACEIs), angiotensin receptor blockers (ARBs), and spironolactone were prescribed following ESC guidelines (10). Follow-up evaluations were scheduled at 7 days and 30 days post-discharge, followed by monthly outpatient visits.

### 2.5 Outcome measures

The primary endpoint of the study was the occurrence of major adverse cardiac and cerebral events (MACCE), including cardiovascular mortality, non-fatal myocardial infarction, non-fatal cerebrovascular events, and ACS requiring hospitalization. Secondary outcomes included post-procedure complications such as contrast-induced nephropathy (CIN), bleeding, hematoma, intensive care unit (ICU) admission, hospital stay duration, and procedural duration.

### 2.6 Statistical analysis

All statistical analyses were performed using IBM SPSS Statistics version 26.0 (IBM Corp., Armonk, NY, USA). The normality of continuous variables was assessed using the Shapiro–Wilk test. Variables following a normal distribution were expressed as mean ± standard deviation (SD) and compared using the independent-samples *t*-test. Non-normally distributed variables were presented as median with interquartile range (IQR) and compared using the Mann–Whitney *U* test. Categorical variables were summarized as frequencies and percentages and compared using the Chi-square test or Fisher's exact test, as appropriate. For comparisons involving more than two groups, the one-way ANOVA, Kruskal–Wallis test, or Fisher–Freeman–Halton exact test was used based on the data distribution. When multiple comparisons were conducted, Bonferroni correction was applied to adjust for type I error inflation.

Multivariate logistic regression analyses were performed to identify independent predictors of MACCE during hospitalization, at 30 days, and at 1 year. Variables with a *p*-value <0.1 in univariate analysis or those considered clinically relevant were included using a backward stepwise elimination method. To minimize overfitting, an events-per-variable ratio >10 was ensured. Multicollinearity was assessed using variance inflation factor (VIF), and model assumptions were verified before inclusion. Model calibration was assessed using the Hosmer–Lemeshow goodness-of-fit test, and explanatory power was measured using Nagelkerke's pseudo-R^2^.

## 3 Result

### 3.1 Patient characteristics

In Vietnam, the COVID-19 pandemic unfolded in four distinct waves, each characterized by unique viral variants and evolving challenges. The first wave, spanning January to April 2020, was associated with the Wuhan variant. The second wave, from July to December 2020, was driven by the D614G variant. The third wave, occurring between January and March 2021, corresponded to the emergence of the Alpha variant. The fourth and most prolonged wave, extending from April 2021 to early 2022, was marked by the predominance of the Delta and Omicron variants (11).

This study included 118 ACS patients who underwent PCI, comprising 26 COVID-19-positive and 92 non-COVID patients. Baseline characteristics such as age, sex, body mass index, and cardiovascular risk factors were comparable between groups. Vital signs were generally similar, except for lower oxygen saturation in the COVID-19 group (94.9 ± 3.2% vs. 98.6 ± 2.2%; *p* = 0.002). COVID-19-positive patients also exhibited significantly higher inflammatory and thrombotic markers, including CRP, white blood cell count, D-dimer, and Troponin I (*p* < 0.01), as well as elevated NT-proBNP. Although left ventricular ejection fraction (LVEF) and estimated glomerular filtration rate (eGFR) trended lower in COVID-19 patients, these differences were not statistically significant. While ACS type distribution was similar between groups, a greater proportion of COVID-19 patients presented with advanced Killip class (≥III: 38.4% vs. 13.0%; *p* = 0.01), indicating more severe heart failure at presentation (Table 1).

**Table 1:** Baseline clinical characteristics of ACS patients undergoing PCI.

Characteristics	Covid 19 *n* = 26	Non-Covid 19 *n* = 92	*p*
Demographics			
Age, years	63.2 ± 8.7	64.3 ± 7.8	0.8*
Male, *n* (%)	15 (57.7%)	55 (59.7%)	0.9^†^
BMI, Kg/m^2^	23.3 ± 3.5	23.4 ± 3.2	0.3*
Hypertension, *n* (%)	12 (45.2%)	41 (44.6%)	0.8^†^
Dyslipidemia, *n* (%)	9 (34.6%)	33 (35.8%)	0.9^†^
CKD, *n* (%)	6 (23.1%)	19 (20.6%)	0.9^†^
Diabetes, *n* (%)	9 (34.6%)	30 (32.6%)	0.8^†^
Smoking, *n* (%)	5 (19.2%)	18 (19.6%)	0.9^†^
Drinking, *n* (%)	5 (19.2%)	20 (21.7%)	0.8^†^
History of CVD, *n* (%)	10 (39.6%)	36 (39.1%)	0.9^†^
Family history of CVD, *n* (%)	4 (13.5%)	17 (18.4%)	0.9^†^
SBP, mmHg	133.5 ± 17.1	133.7 ± 16.2	0.7*
DBP, mmHg	83.3 ± 12.3	83.4 ± 13.4	0.9*
HR, bpm	77.0 ± 9.5	75.4 ± 9.5	0.12*
SpO_2_, %	94.9 ± 3.2	98.6 ± 2.2	**0** **.** **002** *
Laboratory Parameters			
WCB, K/µl	13.9 ± 3.2	11.0 ± 2.8	**0** **.** **005** *
CRP, mg/L	34.1 ± 9.1	11.1 ± 4.3	**<0.001** *
cTnI hs, ng/ml	16.3 ± 5.3	15.3 ± 5.2	**0** **.** **03** *
NT-pro BNP, pg/ml	1,200 [300–1,400]	700 [250–1,100]	**0** **.** **003** **^‡^**
D-Dimer, mg/L	1.1 ± 0.3	0.7 ± 0.2	**<0.001** *
Creatinine, μmol/L	85.8 ± 14.8	82.6 ± 13.0	0.3*
eGFR, ml/min	66.7 ± 11.0	70.4 ± 11.6	0.06*
LV ejection fraction, %	51.3 ± 5.7	54.7 ± 6.1	0.2*
COVID 19 vaccination, *n* (%)	7 (26.9)	36 (39.1)	0.4^†^
COVID-19 Presentation			
Diagnosis at presentation, *n* (%)			0.9^†^
STEMI	16 (61.5)	59 (64.1)	
NSTEMI	6 (23.1)	20 (21.8)	
UA	4 (15.4)	13 (14.1)	
Killip classification, *n* (%)			**0** **.** **01** **^†^**
1	11 (42.4)	61 (66.3)	
2	5 (19.2)	19 (20.7)	
3	5 (19.2)	9 (9.7)	
4	5 (19.2)	3 (3.3)	

* *t*-test.

^†^ Chi-square test.

^‡^ Mann–Whitney *U* test.

Values are *n* (%), mean ± SD, median (IQR).

The bold values represent statistically significant results.

BMI, Body mass index; CKD, chronic kidney disease; CVD, cardiovascular disease; SBP, systolic blood pressure; DBP, diastolic blood pressure; HR, heart rate; SpO_2_, saturation of peripheral oxygen; WCB, white blood cell count; CRP, C-reactive protein; cTnI, cardiac Troponin I; NT-pro BNP, N-terminal pro B-type natriuretic peptide; eGFR, estimated glomerular filtration rate; LV, left ventricular; STEMI, ST-elevation myocardial infarction; NSTEMI, Non-ST-elevation myocardial infarction; UA, unstable angina.

Analysis of clinical trends across successive pandemic waves revealed a progressive escalation in disease severity. Patients in Waves 1 and 2 demonstrated relatively stable inflammatory profiles, with white blood cell (WBC) counts comparable to those observed in non-COVID-19 individuals. In contrast, Waves 3 and 4 exhibited significantly elevated WBC counts (Wave 3: 14.1 ± 3.9; Wave 4: 15.4 ± 2.5 K/µl; *p* < 0.01) and modestly increased D-dimer levels (Wave 4: 1.2 ± 0.4 mg/L vs. Wave 1: 1.0 ± 0.4 mg/L; *p* = 0.7). These changes were paralleled by a rise in the proportion of patients requiring oxygen therapy or admission to the intensive care unit (ICU). The prevalence of Killip class III–IV heart failure increased markedly, affecting over 50% of patients in Wave 4 compared to only 14.3% in Wave 1. Although comorbidities and demographic variables remained consistent, the cumulative clinical burden—including cardiogenic shock and reduced LVEF—was more pronounced in the later waves. These findings reflect an evolving clinical phenotype, with later waves associated with more severe inflammation, thrombotic activity, and hemodynamic compromise (Table 2, Supplementary Table S1).

**Table 2:** Severity of COVID-19 at admission among ACS patients.

Characteristics	Covid 19 Wave 1 *n* = 7	Covid 19 Wave 2 *n* = 5	Covid 19 Wave 3 *n* = 6	Covid 19 Wave 4 *n* = 8	*p*
COVID 19 severity, *n* (%)					**0.01** **^†^**
Mild	2 (28.6%)	2 (40%)	1 (16.7%)	1 (12.5%)	
Moderate	2 (28.6%)	1 (20%)	1 (16.7%)	1 (12.5%)	
Severe	2 (28.6%)	1 (20%)	2 (33.3%)	3 (37.5%)	
Critical	1 (14.2%)	1 (20%)	2 (33.3%)	3 (37.5%)	
Oxygen treatment, *n* (%)					**<0.01** **^†^**
None	2 (28.6%)	3 (60%)	1 (16.7%)	1 (12.5%)	
Cannula	2 (28.6%)	1 (20%)	1 (16.7%)	1 (12.5%)	
Mask	2 (28.6%)	1 (20%)	1 (16.7%)	1 (12.5%)	
HFNC	1 (14.3%)	0 (0%)	1 (16.7%)	2 (25%)	
Intubated	0 (0%)	0 (0%)	2 (33.2%)	3 (37.5%)	
Corticoid treatment, *n* (%)	0 (0%)	1 (20%)	3 (50%)	5 (62.5%)	**<0.01** **^†^**

^†^ Fisher–Freeman–Halton Exact Test.

Values are *n* (%).

The bold values represent statistically significant results.

HFNC, High-flow nasal cannula.

### 3.2 Coronary lesions and intervention details

Procedural features were largely comparable between COVID-19 and non-COVID-19 patients. Radial access was predominant in both groups (80.8% vs. 90.2%; *p* = 0.3), with no significant differences in PCI duration, contrast volume, or fluoroscopy time. The LAD was the most common culprit vessel in both group (*p* = 0.6). Multivessel disease was more frequent in the COVID-19 group (26.9% vs. 13.1%; *p* = 0.4), while thrombus burden was higher (grade ≥3: 42.3% vs. 16.3%; *p* = 0.01). Poor baseline TIMI flow (0–I) was more common in COVID-19 patients (61.5% vs. 43.4%; *p* = 0.1), although final TIMI 3 flow was achieved in most cases (92.3% vs. 100%; *p* = 0.1). Myocardial blush grade ≥2 and complete lesion resolution were slightly lower in the COVID-19 group (both 92.3% vs. 100%; *p* = 0.07). Procedural success remained high (92.3% vs. 100%; *p* = 0.5), with no significant differences in arrhythmias or no-reflow events (*p* = 0.5). The only significant difference was a higher heparin dose in COVID-19 patients (131.5 ± 30.6 vs. 125.7 ± 19.4 U/kg; *p* = 0.03) (Table 3).

**Table 3:** Procedural characteristics.

Characteristics	Covid 19 *n* = 26	Non-Covid 19 *n* = 92	*p*
Radial access, *n* (%)	21 (80.8)	83 (90.2)	0.3^†^
Femoral access, *n* (%)	5 (19.2)	9 (9.8)	0.3^†^
Duration of case, min	51.8 ± 13.6	49.0 ± 11.5	0.9*
Culprit vessel, *n* (%)			0.6^‡^
LM	4 (15.4)	10 (10.9)	
LAD	17 (65.4)	51 (55.4)	
LCX	3 (11.5)	20 (21.7)	
RCA	5 (19.2)	21 (22.8)	
≥2 vessels disease, *n* (%)	7 (26.9)	12 (13.1)	0.4^†^
Thrombus burden ≥3, *n* (%)	11 (42.3)	15 (16.3)	**0** **.** **01** **^†^**
Baseline TIMI, *n* (%)			0.1^‡^
0–I	16 (61.5)	40 (43.4)	
II	10 (38.5)	43 (46.7)	
III	0	9 (9.9)	
TIMI after stenting			0.1^‡^
0–I	0	0	
II	2 (7.7)	0	
III	24 (92.3)	92 (100)	
Post-PCI myocardial blush grade 2–3	24 (92.3)	92 (100)	0.07^†^
Residual stenosis ≥30%, *n* (%)	2 (7.7)	0	0.07^‡^
Procedural success, *n* (%)	24 (92.3)	92 (100)	0.5^†^
Complication during procedure			0.5^‡^
Vessel compromise	0	0	
Dissection	0	0	
Tamponade	0	0	
Arrhythmia	4 (15.4)	7 (7.6)	
No-reflow phenome	1 (3.8)	0	
Contrast volume, ml (mean ± SD)	158.1 ± 49.3	157.4 ± 47.6	0.1*
Total fluoroscopy time, min (mean ± SD)	21.4 ± 7.2	20.5 ± 6.9	0.1*
Total heparin dose per weight, U/kg	131.5 ± 30.6	125.7 ± 19.4	**0** **.** **03** *

* *t*-test.

^†^ Chi-square test*.*

^‡^ Fisher exact test*.*

Values are *n* (%), mean ± SD, median (IQR).

The bold values represent statistically significant results.

LM, left main coronary; LAD, left anterior descending artery; LCX, left circumflex coronary artery; RCA, right coronary artery.

Across the four COVID-19 waves (Supplementary Table S2), procedural patterns suggested increasing complexity. Radial access continued to be the predominant approach (71.4–87.5%; *p* = 0.5), while the LAD was the leading culprit vessel across all waves. PCI duration rose from 45.6 ± 10.3 min in Wave 1 to 56.3 ± 18.8 min in Wave 4 (*p* = 0.4), paralleled by increased thrombus burden (grade ≥3: 28.6%–50%) and more frequent baseline TIMI ≤ I (62.5% in Wave 4). Myocardial blush grade 2–3 declined slightly in Waves 1 and 4 (85.7% and 87.5%). Heparin dose increased across waves, peaking in Wave 4 (143.6 ± 28.2 U/kg; *p* = 0.03). Despite the more complex presentations, procedural success remained ≥85% across all waves. Arrhythmias and no-reflow phenomena were infrequent overall but appeared more often in later waves, reflecting evolving thrombo-inflammatory burden.

### 3.3 Clinical outcomes

COVID-19 patients experienced significantly worse in-hospital outcomes, including higher ICU admission (42.3% vs. 7.6%) and longer hospital stays (9.7 ± 3.7 vs. 6.5 ± 2.5 days; both *p* < 0.001). Although not statistically significant, rates of contrast-induced nephropathy (19.2% vs. 9.8%), major bleeding (7.7% vs. 2.2%), stroke (7.7% vs. 1.1%; *p* = 0.06), and in-hospital mortality (11.5% vs. 5.4%) were numerically higher, contributing to increased MACCE (19.2% vs. 6.5%; *p* = 0.05). At 30 days, COVID-19 patients had higher mortality (23.1% vs. 7.6%; *p* = 0.02) and MACCE (30.8% vs. 9.8%; *p* = 0.01), with these differences persisting at 1 year (MACCE: 42.3% vs. 19.6%; *p* = 0.02). Mortality at 1 year was higher but not significant (23.1% vs. 10.9%; *p* = 0.1). Revascularization and stroke remained more frequent but without statistical significance (Table 4).

**Table 4:** Outcomes of PCI-treated ACS patients during the pandemic.

Characteristics	Covid 19 *n* = 26	Non-Covid 19 *n* = 92	*p*
In-hospital outcomes			
CIN, *n* (%)	5 (19.2)	9 (9.8%)	0.2^†^
ICU admission	11 (42.3)	7 (7.6%)	**<0.001** **^†^**
Hospital length of stay (days)	9.7 ± 3.7	6.5 ± 2.5	**<0.001** *
Major bleeding, *n* (%)	2 (7.7)	2 (2.2%)	0.2^‡^
Minor bleeding, *n* (%)	3 (11.5)	3 (3.3%)	0.08^‡^
In-hospital mortality, *n* (%)	3 (11.5)	5 (5.4%)	0.3^‡^
Stroke, *n* (%)	2 (7.7)	1 (1.1%)	0.06^‡^
Myocardial reinfarction, *n* (%)	0	0	–
MACCE, *n* (%)	5 (19.2)	6 (6.5%)	0.05^†^
30-Day Outcomes			
Mortality, *n* (%)	6 (23.1)	7 (7.6%)	0.02^†^
Stroke, *n* (%)	2 (7.7)	2 (2.2%)	0.2^‡^
Revascularization, *n* (%)	0	0	–
MACCE, *n* (%)	8 (30.8)	9 (9.8%)	**0** **.** **01** **^†^**
1-Year Outcomes			
Mortality, *n* (%)	6 (23.1)	10 (10.9%)	0.1^†^
Stroke, *n* (%)	2 (7.7)	3 (3.3%)	0.3^‡^
Revascularization, *n* (%)	3 (11.5)	5 (5.4%)	0.2^‡^
MACCE, *n* (%)	11 (42.3)	18 (19.6%)	**0** **.** **02** **^†^**

* *t*-test.

^†^ Chi-square test.

^‡^ Fisher exact test*.*

Values are *n* (%) and mean ± SD.

The bold values represent statistically significant results.

CIN, contrast-induced nephropathy; ICU, intensive care unit; MACCE, major adverse cardiac and cerebrovascular event.

Outcomes among COVID-19 patients worsened across pandemic waves. ICU admissions rose from 14.3% in Wave 1 to 62.5% in Wave 4 (*p* < 0.01), with hospital stays increasing accordingly (7.3 ± 2.1 to 12.4 ± 4.2 days; *p* < 0.01). MACCE incidence increased in-hospital (0%–37.5%), at 30 days (14.3%–50%), and at 1 year (14.3%–62.5%; all *p* < 0.01), reflecting greater disease severity in later waves. Mortality followed a similar trend but was not statistically significant (Supplementary Table S3).

**Figure 1:**
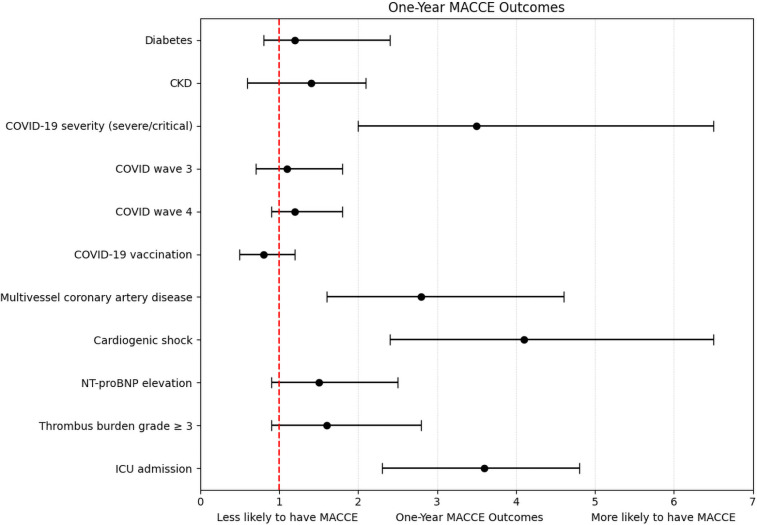
Forest plot of multivariate analysis for one-year MACCE outcomes.

A total of 22 in-hospital MACCE events, 26 MACCE events at 30 days, and 30 events at 1-year follow-up were included in the respective logistic regression models. Severe COVID-19, cardiogenic shock, ICU admission, and multivessel disease consistently emerged as independent predictors across all timepoints (ORs: 2.6–4.1; all *p* < 0.001). Other variables, including diabetes, CKD, elevated biomarkers, vaccination status, and pandemic wave, were not significant (Table 5, Figure 1). The models demonstrated good calibration (Hosmer–Lemeshow *p* > 0.05) and modest explanatory power (pseudo-R^2^: 0.18–0.22) (Supplementary Table S4, Figure S1).

**Table 5:** Multivariate analysis of factors affecting MACCE outcomes after PCI.

Independent variables	In-hospital MACCE		30-day MACCE		One-year MACCE	
	OR (95% CI)	*P* value	OR (95% CI)	*P* value	OR (95% CI)	*P* value
Diabetes	1.2 (0.8–1.9)	0.2	1.3 (0.9–2.1)	0.1	1.2 (0.8–2.4)	0.1
CKD	1.3 (0.9–2.1)	0.1	1.2 (1.0–2.3)	0.09	1.4 (0.6–2.1)	0.3
COVID-19 severity (severe/critical)	3.2 (2.1–4.9)	**<0** **.** **001**	3.4 (2.3–5.2)	**<0** **.** **001**	3.5 (2.0–6.5)	**<0** **.** **001**
Covid wave						
3	1.1 (0.8–1.8)	0.3	1.2 (0.9–2.2)	0.3	1.1 (0.7–1.8)	0.3
4	1.2 (0.9–2.4)	0.2	1.3 (1.0–2.2)	0.07	1.2 (0.9–1.8)	0.1
Covid 19 vaccination	0.8 (0.5–1.3)	0.4	0.9 (0.6–1.5)	0.4	0.8 (0.5–1.2)	0.4
Multivessel coronary artery disease	2.6 (1.7–4.1)	**<0** **.** **001**	2.7 (1.8–4.4)	**<0** **.** **001**	2.8 (1.6–4.6)	**<0** **.** **001**
Cardiogenic shock	3.7 (2.3–5.9)	**<0** **.** **001**	3.8 (2.5–6.2)	**<0** **.** **001**	4.1 (2.4–6.5)	**<0** **.** **001**
NT-proBNP elevation	1.4 (0.8–2.4)	0.2	1.5 (1.0–2.6)	0.08	1.5 (0.9–2.5)	0.1
Thrombus burden grade ≥3	1.6 (0.9–2.6)	0.1	1.7 (1.0–2.9)	0.07	1.6 (0.9–2.8)	0.2
ICU admission	2.8 (1.8–4.5)	**<0** **.** **001**	3.4 (1.8–4.8)	**<0** **.** **001**	3.6 (2.3–4.8)	**<0** **.** **001**

The bold values represent statistically significant results.

## 4 Discussion

COVID-19 presents considerable challenges in managing patients with ACS due to its hyperinflammatory and pro-thrombotic state, which exacerbates pre-existing cardiovascular conditions (12). The virus induces endothelial dysfunction, platelet activation, and cytokine storm, ultimately contributing to plaque destabilization, thrombus formation, and increased cardiovascular risks (13, 14). These systemic effects further aggravate underlying coronary artery disease, complicating PCI procedures and increase the risk of severe complications such as arrhythmias, cardiogenic shock, no-reflow phenomena, and coronary artery re-occlusion (15).

In this study, we assessed PCI outcomes of ACS patients with and without COVID-19 infection during four distinct pandemic waves in Vietnam. Although baseline characteristics were similar, COVID-positive patients exhibited more severe clinical and laboratory profiles, including lower oxygen saturation, higher inflammatory and thrombotic markers, and an elevated incidence of cardiogenic shock. Coronary angiography revealed a markedly higher thrombus burden among COVID-positive patients These findings support existing evidence of COVID-19-induced vascular inflammation and thrombosis that contribute to adverse outcomes (14, 16). Despite these challenges, primary PCI achieved comparable reperfusion rate in both groups.

However, even with procedural success, COVID-positive patients still experienced worse clinical outcomes, including increased ICU admissions, longer hospital stays, and higher rates of in-hospital mortality and stroke. This trend was especially pronounced during Wave 4, dominated by the Delta variant, which was associated with the most severe clinical presentations, highest thrombus burden, and elevated 1-year MACCE rate (62.5%, *p* = 0.02). Delta variant is known for its increased transmissibility and its ability to trigger a more intense inflammatory and pro-thrombotic response compared to earlier variants (17, 18). Multivessel disease and cardiogenic shock were also more common in COVID-19 patients, which may have further contributed to poor prognosis despite timely PCI.

Many large-scale studies from high-income countries have documented poorer in-hospital outcomes in ACS patients with concurrent COVID-19. For instance, a study by Markson et al. (19) found significantly higher incidences of hospital mortality, cardiac arrest, cardiogenic shock, and respiratory failure in ACS patients with COVID-19 compared to non-COVID counterparts (19). Similarly, Krishnaraj S. Rathod's study demonstrated that STEMI patients with COVID-19 were associated with higher rates of cardiac arrest, larger thrombus burdens, more extensive infarctions, and worse clinical outcomes (19). A systematic review by Nicholas W. S. Chew emphasized substantial delays in door-to-balloon time during the pandemic and identified higher in-hospital mortality rates (OR = 1.27; 95% CI: 1.09–1.49). Subgroup analysis revealed that low- and middle-income countries experienced a significantly higher mortality rate during the pandemic, while high-income nations showed a similar trend but did not reach statistical significance (18).

In the multivariate analysis, severe or critical COVID-19 infection, multivessel coronary artery disease, cardiogenic shock, and ICU admission emerged as independent predictors of MACCE across all timepoints. The strong relationship between cardiogenic shock and adverse outcomes is consistent with existing evidence, likely attributable to systemic hypoperfusion, myocardial injury, and inflammatory dysregulation (20). Similarly, ICU admission may serve as a marker of overall disease severity and is frequently accompanied by complications such as ventilator-associated pneumonia or acute kidney injury. Notably, conventional predictors such as NT-proBNP and diabetes did not show statistical significance. This may reflect the overwhelming impact of acute inflammatory and thrombotic processes and COVID-specific complications, which could overshadow the contribution of chronic comorbidities, particularly in smaller cohorts.

These findings add to the limited body of evidence from Southeast Asia, highlighting the distinct healthcare approaches and challenges encountered in Vietnam during the COVID-19 pandemic. While the country implemented a strong public health response during the early waves of the pandemic, prolonged lockdowns, delayed referral systems, and healthcare worker shortages disrupted the continuity of care, particularly for chronic cardiovascular diseases (21, 22). The situation was particularly critical during the Delta wave, when healthcare systems were overwhelmed, resulting in delayed diagnoses, limited ICU availability, and restricted access to advanced interventional tools (7). These systemic pressures likely contributed to poorer clinical outcomes, independent of patient-level factors. Disruptions in care delivery may also explain the delayed presentation of ACS cases observed in the later pandemic waves.

Our study offers an insight into the interaction between COVID-19 and ACS in a resource-limited setting; however, the findings should be interpreted with caution due to inherent limitations, including its retrospective, single-center design and relatively small sample size. Despite these constraints, the results provide a representative overview of the challenges encountered in ACS management during the pandemic and illustrate key procedural adaptations implemented at a tertiary care center in Vietnam. These observations emphasize the need for flexible and resilient healthcare strategies to sustain essential cardiac services in the face of future public health crises.

### 4.1 Study limitations

This study has several limitations should be considered. First, its retrospective, single-center design limits generalizability. Only ACS patients undergoing PCI were included, potentially introducing selection bias by excluding those managed conservatively or unable to access care, particularly during resource-constrained periods of the pandemic. Strict lockdown policies and overwhelmed healthcare services also contributed to a small sample size, reducing statistical power and increasing the risk of type II error. Second, although multivariate logistic regression was conducted, the limited number of events per variable raises concerns about model robustness. Outcomes were not formally adjudicated, introducing potential misclassification. Additionally, in-hospital variables such as door-to-balloon time, pharmacologic treatments, and ventilatory support were not captured, limiting the analysis of treatment-related influences on outcomes. Third, vaccination status and SARS-CoV-2 variants were not analysed due to incomplete documentation, which may have affected disease severity and clinical outcomes across waves. Intravascular imaging techniques were also not utilized due to resource constraints during the pandemic and their high cost, limiting procedural assessment. Lastly, the findings reflect a tertiary hospital in Ho Chi Minh City and may not represent the broader Vietnamese healthcare context, particularly rural or provincial settings with fewer resources. Regional disparities in PCI access, care quality, and patient follow-up were not examined but may significantly impact outcomes.

Future multicenter studies or a national ACS-PCI registry are warranted to better capture variations in patient characteristics, healthcare access, and outcomes across Vietnam. These efforts would provide more representative data to inform equitable policy and strengthen preparedness for future public health crises.

## 5 Conclusion

This study provides real-world insight into the impact of COVID-19 on PCI outcomes in a Vietnamese tertiary care setting, particularly during the fourth wave dominated by the Delta variant. An increase in thrombotic burden, procedural complexity, and adverse outcomes was observed among COVID-19 patients, especially in later waves. These findings underscore the need for timely risk stratification, optimized procedural planning, and sustained vaccination efforts in managing ACS during public health emergencies. However, due to the study's retrospective design, single-center scope, and limited sample size, the results should be interpreted as exploratory rather than definitive. Larger, multicenter studies are needed to validate these observations and inform broader clinical and policy strategies.

## Data Availability

The original contributions presented in the study are included in the article/Supplementary Material, further inquiries can be directed to the corresponding author.
