# Supplementary material for: Outcomes of percutaneous coronary intervention in COVID-19-positive acute coronary syndrome patients: a retrospective study in Vietnam
Source: Front Cardiovasc Med. 2025 Jul 10;12:1563415. doi: 10.3389/fcvm.2025.1563415 (PMC12286974; doi:10.3389/fcvm.2025.1563415)
Supplement: Supplementary file 1 [file Datasheet1.pdf]

# Authorship change

For changes to the authorship of manuscripts, Frontiers requires that all authors complete and sign the Authorship Change form below. This includes any author(s) being added or removed, as well as all other authors included on the original author list. Signing this form indicates consent to the change(s), and agreement with the new Author Contributions, Conflict of Interest and Acknowledgment sections. Ensure that every section of this form is completed.

## Authorship criteria

In accordance with the International Committee of Medical Journal Editors (ICMJE) guidelines, in order to be included in the author list a contributor must meet all 4 of the following criteria:

- Substantial contributions to the conception or design of the work; or the acquisition, analysis, or interpretation of data for the work; AND
- Drafting the work or revising it critically for important intellectual content; AND
- Final approval of the version to be published; AND
- Agreement to be accountable for all aspects of the work in ensuring that questions related to the accuracy or integrity of any part of the work are appropriately investigated and resolved.

Any contributors who meet fewer than all 4 of the above criteria for authorship must not be listed as authors, but their contributions should be detailed in the Acknowledgments section. Requests to add authors who do not meet all 4 of the above criteria will not be considered.

If you have any questions regarding authorship, contact the Research Integrity team of the relevant journal.

Please note that we cannot proceed with the publication of your manuscript until any outstanding authorship issues have been resolved.

# Completing and signing the form

- Complete and submit an Authorship Change form if you: Add, remove, or change the order of the authors after your article is accepted.
- We only accept forms written in English, signatures can be provided in Arabic and Chinese.
- Complete the form with all article and author information, and new statements (on Page 3), if they have changed.
- Complete the last page of the form with the final author list that will be used in the published article.
- The form must be signed individually, by each author.
- The form should be manually signed or contain valid e-signatures (an electronic version of a signature that can be uniquely identified and linked to the signatory, as shown in the example below).
- Useful link: helpx.adobe.com/acrobat/using/signing-pdfs.html.
- Authors can submit signatures across different files individually if the main information is completed and all signatures are provided.

## Acceptable signatures

- Digital stylus.
- Adobe “drawn” (using the default fonts) and “signed” signatures.
- Signature stamps or seals.
- Handwritten signatures.

Electronic signature

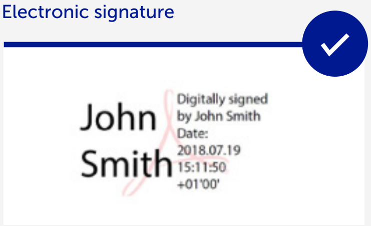


Digital stylus signature

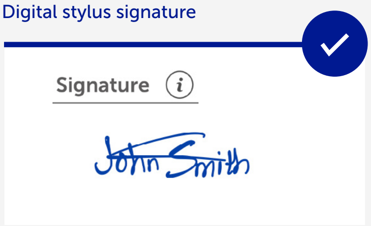


Scanned signature

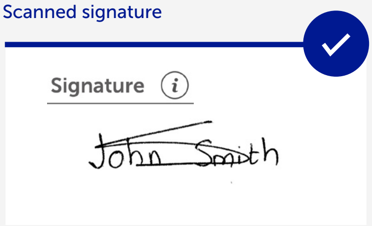


## Unacceptable signatures

- Typed signatures which are not e-signatures will not be accepted.
- Signatures which have been signed on behalf of other authors/not individually signed.
- Photographs of signatures will also not be allowed.

If you provide the form with invalid signatures, we will not be able to accept it or be able to consider your request for an authorship change.

Typed signature

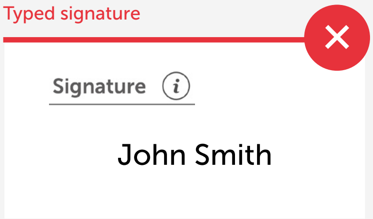


Photograph/screenshot signature

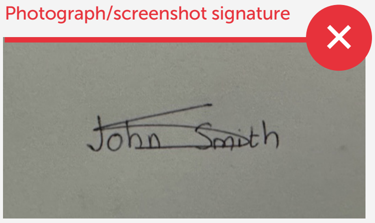

# Authorship change

**对于稿件作者身份的变更，Frontiers要求所有作者填写并签署下方的“作者身份变更表”.任何被添加或移除的作者，以及在原始作者列表中所包含的其他所有作者均需进行填写并签署. 签署此表格即表示同意进行更改，并认可新的作者贡献、利益冲突及致谢部分. 请确保填妥此表格的每个部分.**

## 作者身份标准

根据国际医学期刊编辑委员会（ICMJE）指导方针，文章贡献者须同时符合以下4项标准才能被列入作者名单：

- S对该项工作的构思或设计做出重大贡献；或为工作获取、分析或解析数据；
- 起草文稿或对重要的学术内容进行重大修改；
- 最终批准待发表的版本；
- 同意对该项工作的所有方面负责，以确保与工作的任何部分相关的准确性或完整性问题都能得到适当地调查和解决.

任何不符合上述全部4项作者身份标准的贡献者不得被列为作者，但贡献者的贡献应在致谢部分进行详细说明。不符合上述全部4项标准的作者添加申请将不予考虑.

如果您对作者身份有任何疑问，请随时联系相关期刊的研究诚信团队.

请注意，在任何有关作者身份的问题未得到解决前，我们无法发表您的稿件.

# 填写并签署表格

- 如果您在文章接收后，需要：添加、删除或更改作者的顺序，请填写并提交作者身份变更表.
- 请您用英文填写表格，签名可以是中文.
- 请将所有文章和作者的信息填写在表格中；如果添加或者删除作者，请在第3页填写新的声明.
- 在表格的最后一页填写最终作者名单，该名单将用于在线发表版本中.
- 表格必须由每位作者单独签名.
- 表格必须提供手写签名或包含有效的电子签名（可以唯一识别并链接到签名人的电子版签名，如下例所示）.
- 有效的链接：helpx.adobe.com/acrobat/using/signing-pdfs.html.
- 如果作者因地域原因不能同时在同一个文件签名，可分别在不同文件填写完整信息，并单独完成签名.

## 可接受的签名

- 数字化签名.
- Adobe（使用默认字体）"绘制（drawn）"和"签署（signed）"的签名.
- 印章签名.
- 手写签名.

Electronic signature

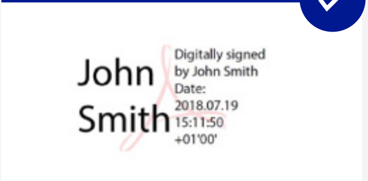


Digital stylus signature

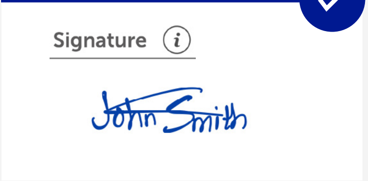


Scanned signature

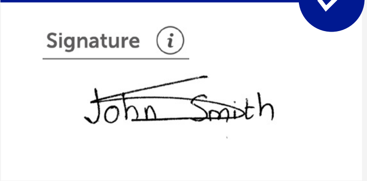


## 不可接受的签名

- 不接受非电子签名的打字签名.
- 代表其他作者签署的签名/非独立完成的签名.

如果您提供的表格签名无效，我们将无法接受或考虑您更改作者身份的请求

Typed signature

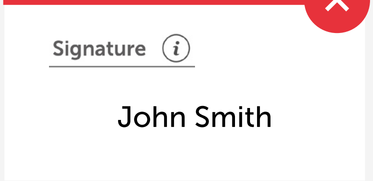


Photograph/screenshot signature

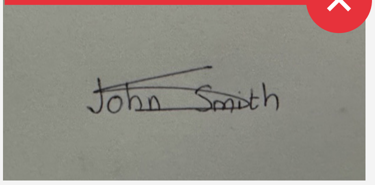

Manuscript ID number

1563415

DOI

Manuscript title

Outcomes of percutaneous coronary intervention in COVID-19-positive acute coronary syndrome patients: A retrospective study in Vietnam

Please tick as appropriate

- ○ Addition of new author(s)
- ● Removal of author(s)
- ○ Addition and removal of author(s)
- ○ Change in the order of author(s)

## Details of author(s) to be added/removed

Complete one table for each author to be added/removed. If you wish to add/remove more than 5 authors, contact the Research Integrity team. Please ensure you provide as much detail as possible in the 'Reason for change' section so we can verify why the change is necessary. This should include details about:

- Why the change is being made.
- Why the author in question was/was not included in the original author list.
- Whether additional work was carried out in response to comments from reviewers and/or editors. If so, please provide details of this work in the 'Individual contributions' section.

If the reason provided is insufficiently detailed or does not address the above points, the Research Integrity team may contact you for additional information. We will be unable to change the author list for your manuscript without sufficient reason being provided.

请为每位需被添加/删除的作者填写单独的申请表格。如果您希望添加/移除5位以上的作者，请您联系相关期刊的研究诚信团队. 请确保在“变更原因”一栏中提供尽可能详细的信息，以便验证作者变更的必要性. 请注意包含以下详细信息：

- 为什么要进行更改.
- 为什么更改的作者在/不在原始作者名单中.
- 对于来自审稿人及编辑的评论是否进行了额外的工作。如果“是”，请在“个人贡献”部分提供该项工作的详细信息.

如果提供的原因不够详细或未解决上述问题，研究诚信团队可能会与您进一步联系已获取更多信息。若无法提供全面且详细的信息，我们将无法进行该稿件作者名单的更改.

First name
Hoa

Surname
The

Email
Bui

Organization name
Department of Cardiovascular Intervention, Nguyen Tri Phuong Hospital, Ho Chi Minh city

**Addition/removal** tick as appropriate
○ Addition ● Removal

Individual contributions
supervision and methodology

Reason for change
I acknowledge that my involvement in the project did not meet the authorship criteria as outlined by the ICMJE guidelines.
Additionally, I recognize that a potential conflict of interest related to my recent professional affiliations may give rise to concerns regarding the objectivity of the publicatkion

Signature
Only for authors being removed.
Authors being added should sign page 9:

Digitally signed by The Hoa
DN: CN=The Hoa, E=hoabtcardio@yahoo.com
Reason: I am the author of this document
Foxit PDF Reader Version: 2024.2.0

First name
Duy

Surname
Le Cao Phuong

Email
pduycardio@gmail.com

Organization name
Department of Cardiovascular Intervention, Nguyen Tri Phuong Hospital, Ho Chi Minh city

**Addition/removal** tick as appropriate
○ Addition ○ Removal

Individual contributions
Conceptualization, formal analysis, investigation, methodology;project administration, resources:, supervision, writing-review and editing

Reason for change
Dr. Hoa The Bui is being removed from the author list due to limited contribution that did not meet ICMJE authorship criteria. Additionally, recently a potential conflict of interest related to his professional affiliation was identified. To ensure transparency and uphold the integrity of the publication, Dr. Bui will be acknowledged for his early involvement instead.

Signature
Only for authors being removed.
Authors being added should sign page 9:

Digitally signed by Le Cao Phuong Duy
DN: OU=Nguyen Tri Phuong Hospital, CN=Le Cao Phuong Duy, E=pduycardio@gmail.com
Reason: I am the author of this document

frontiers

First name
Nguyet

Surname
Nguyen Thi Minh

Email
minhnguyetcardio@gmail.com

Organization name
Department of Cardiovascular Intervention, Nguyen Tri Phuong Hospital, Ho Chi Minh city

**Addition/removal** tick as appropriate

Addition    Removal

Individual contributions

formal analysis, funding acquisition, investigation, methodology, project administration, software, writing-review and editing

Reason for change

Dr. Hoa The Bui is being removed from the author list due to limited contribution that did not meet ICMJE authorship criteria. Additionally, recently a potential conflict of interest related to his professional affiliation was identified. To ensure transparency and uphold the integrity of the publication, Dr. Bui will be acknowledged for his early involvement instead.

Signature
Only for authors being removed.
Authors being added should sign page 9:

Nguyen Thi Minh Nguyet

Digitally signed by Nguyen Thi Minh Nguyet
DN: OU=Nguyen Tri Phuong Hospital, CN=Nguyen Thi Minh Nguyet, E=minhnguyetcardio@gmail.com
Reason: I am approving this document

First name
Quan

Surname
Vo Duy

Email
dr.duyquan@gmail.com

Organization name
Nguyen Tat Thanh university

**Addition/removal** tick as appropriate

Addition    Removal

Individual contributions

Conceptualization, data curation, software, validation, visualization, writing-original draft preparation, writing-review and editing

Reason for change

Dr. Hoa The Bui is being removed from the author list due to limited contribution that did not meet ICMJE authorship criteria. Additionally, recently a potential conflict of interest related to his professional affiliation was identified. To ensure transparency and uphold the integrity of the publication, Dr. Bui will be acknowledged for his early involvement instead.

Signature
Only for authors being removed.
Authors being added should sign page 9:

Digitally signed by Vo Duy Quan
DN: OU=Nguyen Tat Thanh University, CN=Vo Duy Quan, E=dr.duyquan@gmail.com
Reason: I am approving this document
Date: 2025.06.09 16:38:02+09'00'
Foxit PDF Reader Version: 2024.2.0

frontiers

First name

Surname

Email

Organization name

**Addition/removal** tick as appropriate

Addition

Removal

Individual contributions

Reason for change

Signature

Only for authors being removed.
Authors being added should sign page 9:

First name

Surname

Email

Organization name

**Addition/removal** tick as appropriate

Addition

Removal

Individual contributions

Reason for change

Signature

Only for authors being removed.
Authors being added should sign page 9:

## Provide the author list in the order that you would like it to be published and with the correct spelling.

请按照文章发表时您所希望呈现的作者顺序提供相应的作者名单及作者姓名的正确拼写.

| | First name(s) | Surname | Email address | Signature | Date |
|---|---|---|---|---|---|
| 01 | Duy | Cao Phương Le | pduycardio@gmail.com | Digitally signed by Le Cao Phuong Duy DN: OU=Nguyen Tri Phuong Hospital, CN=Le Cao Phuong Duy, E=pduycardio@gmail.com Reason: I am the author of this document | 09/06/2025 |
| 02 | Nguyet | Thi Minh Nguyen | minhnguyetcardio@gmail.com | Nguyễn Thị Minh Nguyệt Digitally signed by Nguyen Thi Minh Nguyet DN: OU=Nguyen Tri Phuong Hospital, CN=Nguyen Thi Minh Nguyet, E=minhnguyetcardio@gmail.com Reason: I am approving this document | 09/06/2025 |
| 03 | Quan | Duy Vo | dr.duyquan@gmail.com | Digitally signed by Vo Duy Quan DN: OU=Nguyen Tat Thanh University, CN=Vo Duy Quan, E=dr.duyquan@gmail.com Reason: I am approving this document Date: 2025.06.09 16:37:46+09'00' Foxit PDF Reader Version: 2024.2.0 | 09/06/2025 |
| 04 | | | | | |
| 05 | | | | | |
| 06 | | | | | |
| 07 | | | | | |
| 08 | | | | | |
| 09 | | | | | |
| 10 | | | | | |
| 11 | | | | | |
| 12 | | | | | |
| 13 | | | | | |
| 14 | | | | | |
| 15 | | | | | |
| 16 | | | | | |
| 17 | | | | | |
| 18 | | | | | |
| 19 | | | | | |
| 20 | | | | | |
| 21 | | | | | |
| 22 | | | | | |
| 23 | | | | | |
| 24 | | | | | |
| 25 | | | | | |
